# Regarding Iannicelli et al.

**DOI:** 10.1186/s13052-021-01009-4

**Published:** 2021-03-27

**Authors:** Evelyn Chan, Paul Leong

**Affiliations:** School of Clinical Sciences, Monash Health, 246 Clayton Rd, Clayton, Victoria Australia

Dear Professor Corsello,

We read with interest the Review published by Iannicelli and colleagues [1] which reviewed our publication [2].

We write to clarify one point. The Review states that “the sample size analyzed was not numerically sufficient in the statistical analysis”. We respectfully differ on this conclusion.

We performed two studies (*n* = 123 and *n* = 131 respectively), both of which exceeded the a priori power calculation required of 114 patients per clinical setting. Indeed, pointed out in Table 1 of Iannicelli et al., our study sample size is by far the largest in the literature. This is reflected in statistically significant findings in the primary endpoint of change from baseline pain (with *P* = 0.018 and *P* = 0.034 respectively).

It is unclear how Iannicelli and colleagues came to regard the sample size as insufficient when our study is the largest in the literature, met a priori study recruitment goals, and formal statistical tests confirm significance.

Yours sincerely

Dr. Evelyn Chan and Dr. Paul Leong on behalf of the authors

## Data Availability

Not applicable.
